# Prostatic Solitary Fibrous Tumor With Pulmonary Metastases: A Case Report

**DOI:** 10.7759/cureus.94849

**Published:** 2025-10-18

**Authors:** Yunus Kaygusuz, Fatih Kus, Feride Yilmaz, Mujdat Ayva, Olcay Kurtulan, Gunes Guner, Ahmet Gudeloglu, Mustafa Erman

**Affiliations:** 1 Internal Medicine, Hacettepe University, Ankara, TUR; 2 Oncology, Hacettepe University, Ankara, TUR; 3 Urology, Hacettepe University, Ankara, TUR; 4 Pathology, Hacettepe University, Ankara, TUR; 5 Medical Oncology, Hacettepe University, Ankara, TUR

**Keywords:** holmium laser enucleation of the prostate (holep), isolated lung metastasis, malignant solitary fibrous tumor, stat6, tumour of prostate

## Abstract

Solitary fibrous tumors (SFT) are rare intermediate-grade mesenchymal neoplasms with unpredictable clinical courses, including the potential for late metastases. While most commonly arising in the pleura, extrapleural SFTs have been described. However, SFTs originating from the prostate are exceptionally rare, and their potential for metastatic progression remains largely unknown. A 59-year-old male presented with lower urinary tract symptoms and underwent Holmium laser enucleation of the prostate (HoLEP) after unsuccessful medical therapy. Histopathological examination revealed spindle-shaped neoplastic cells that were positive for cluster of differentiation 34 (CD34) and signal transducer and activator of transcription 6 (STAT6), confirming SFT. Radical prostatectomy was subsequently performed with negative surgical margins. Twenty-three months later, thoracic imaging detected two pulmonary nodules. Fibroblast activation protein inhibitor (FAPI) positron emission tomography (PET) and percutaneous biopsy confirmed metastatic SFT. Both lung lesions were surgically resected, followed by adjuvant chemotherapy with ifosfamide and doxorubicin. The patient has remained in remission for 18 months. This case emphasizes that even histologically benign-appearing prostatic SFTs can demonstrate malignant potential. It also underscores the necessity of long-term surveillance and contributes to the literature by reporting the first known case of a metastatic SFT originating from the prostate gland.

## Introduction

Solitary fibrous tumors (SFT) are exceptionally rare fibroblastic mesenchymal neoplasms, generally characterized by intermediate malignant potential and a low propensity for metastasis [1]. Its age-adjusted incidence is approximately 1 per 100,000 individuals [2], representing 2%-4% of all soft tissue tumors [3,4]. Initially described in the pleura, SFTs have since been reported at various anatomical locations. The median age at diagnosis typically falls within the fifth or sixth decade of life [2].

Complete surgical resection is the mainstay of treatment for localized SFTs [2]. Histopathological features suggestive of malignancy include tumor size greater than 10 cm, mitotic rate exceeding four mitoses per 10 high-power fields (HPFs), presence of necrosis, and nuclear polymorphism. However, there is considerable biological unpredictability, as tumors classified histologically as benign may still metastasize during follow-up, whereas some tumors deemed malignant may not [5]. Immunohistochemical (IHC) markers such as cluster of differentiation 34 (CD34), B-cell lymphoma-2 (Bcl-2), and signal transducer and activator of transcription 6 (STAT6) are instrumental in diagnosis [6].

Given the potential for late recurrence, long-term surveillance is critical; metastasis rates can approach 50% over a 20-year follow-up period [7]. Prostatic SFTs are extraordinarily rare, with fewer than 40 cases reported in the literature to date [8]. To our knowledge, no cases of metastatic SFT originating from the prostate have been reported previously.

Herein, we present a unique case of prostatic SFT, initially managed surgically, that subsequently developed into pulmonary metastases approximately two years after diagnosis. To the best of our knowledge, this is the first reported case of metastatic SFT originating from the prostate gland.

## Case presentation

A 59-year-old male presented to our institution with nocturia and difficulty initiating urination. Uroflowmetry demonstrated an obstructive pattern and a high post-void residual volume. His serum prostate-specific antigen (PSA) level was within normal limits. Transrectal ultrasonography (TRUS) revealed significant prostatomegaly, with a measured volume of 120 mL. Based on these findings, benign prostatic hyperplasia (BPH) was initially considered as the primary diagnosis. Initial medical management with silodosin and dutasteride was initiated; however, after three months of therapy, his symptoms persisted without notable improvement.

Given the large prostate volume, refractoriness to medical therapy, impact of symptoms on the quality of life, and patient preference, surgical intervention was recommended. In such cases of refractory lower urinary tract symptoms despite standard BPH therapy, differential diagnoses such as prostate cancer, prostatic stromal tumor, chronic prostatitis, or other rare prostatic neoplasms should also be considered. Holmium laser enucleation of the prostate (HoLEP) was performed. Enucleation was completed in 40 minutes; however, due to the unusually firm consistency of the prostatic tissue, morcellation, which typically requires approximately 20 minutes for prostates of this size at our center, was notably prolonged, taking 315 minutes across three consecutive sessions over three days.

**Figure 1:**
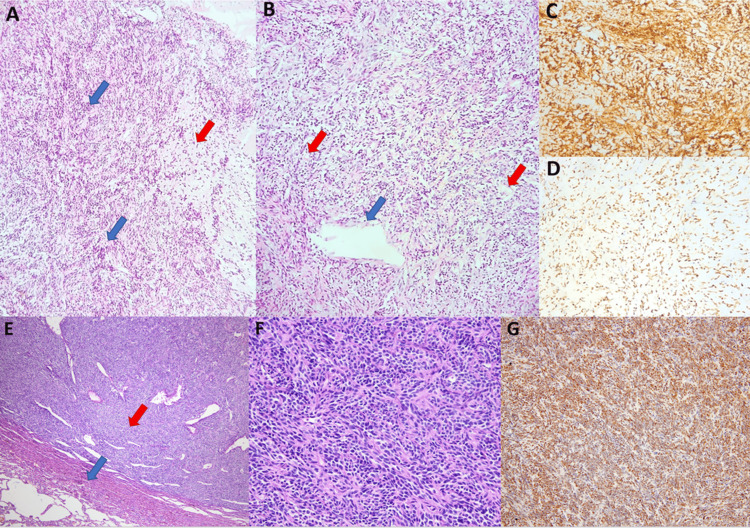
Histopathological and immunohistochemical features of the tumor. Histopathological and immunohistochemical features of the tumor. (A) Fragments of a solid mass obtained by HoLEP, showing alternating hyper- (blue arrows) and hypocellular (red arrow) areas. (B) Variably sized vessels (blue arrow: large vessel, red arrows: small vessels). (C, D) Strong and diffuse positivity for CD34 and STAT6. (E) A well-circumscribed tumor nodule (red arrow) localized in the lung parenchyma (blue arrow) (H&E, ×40). (F) Fascicular arrangement of uniform spindle cells (H&E, ×200). (G) Immunohistochemical STAT6 positivity (×100). HoLEP, Holmium laser enucleation of the prostate; STAT6, signal transducer and activator of transcription 6; CD34, cluster of differentiation 34; H&E, hematoxylin and eosin

Histopathological evaluation of the resected specimen revealed spindle-shaped neoplastic cells, focal necrosis, and nuclear pleomorphism. Immunohistochemical analysis revealed diffuse positivity for CD34 and STAT6, with a low mitotic rate (0-1 mitoses per 10 high-power fields) and a Ki-67 proliferation index of 2%-3%. These findings were indicative of a solitary fibrous tumor (Figure 1).

**Figure 2:**
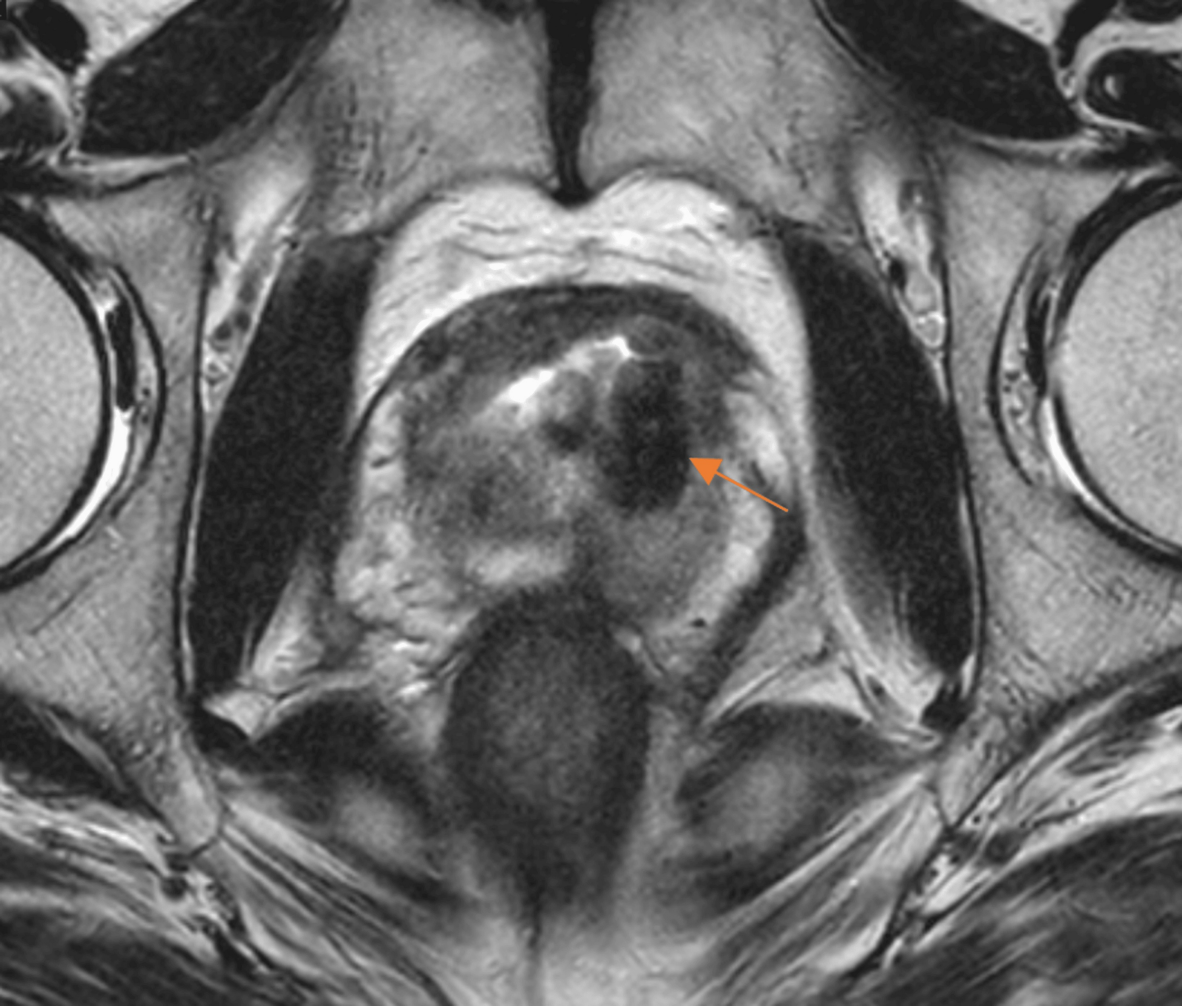
Prostate MRI after HoLEP. T2-weighted multiparametric prostate MRI demonstrating a large defect consistent with sequelae of prior HOLEP. No distinct mass lesion or pathological lymphadenopathy is observed. HoLEP, Holmium laser enucleation of the prostate; MRI, magnetic resonance imaging

Given the potential aggressiveness of SFTs, multiparametric magnetic resonance imaging (MRI) of the prostate was performed, revealing prostatomegaly but no distinct mass lesion or pathological lymphadenopathy (Figure 2).

**Figure 3:**
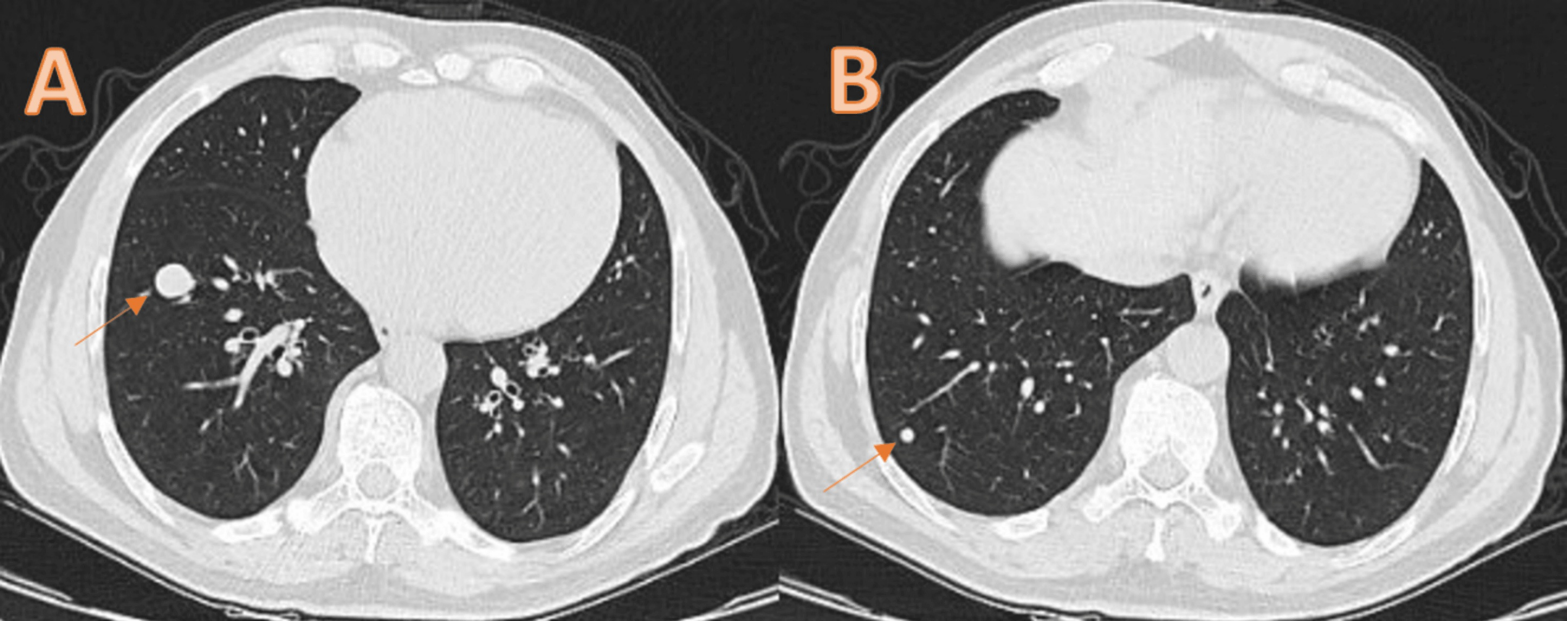
Pulmonary nodules detected on chest CT. (A) The arrow indicates a 14 × 12 mm nodule in the lateral basal segment of the right lower lobe. (B) The arrow indicates a 5 mm nodule in the posterior basal segment of the right lower lobe. CT, computed tomography

In addition, chest and abdominal computed tomography (CT) showed no evidence of distant metastases. The patient subsequently underwent a non-nerve-sparing open radical prostatectomy. The surgical specimen contained a well-circumscribed, cream-colored, fibrillary solid nodule measuring 2 × 1.8 × 1.3 cm with negative surgical margins. The postoperative recovery was uneventful. No adjuvant local or systemic therapies were administered. Routine surveillance imaging revealed two new pulmonary nodules on thoracic CT performed at 23 months postoperatively: a 14 × 12 mm nodule in the right lower lobe lateral basal segment and a 5 mm nodule in the posterior basal segment (Figure 3).

Although SFTs are known for low fluorodeoxyglucose (FDG) avidity, positron emission tomography (PET) imaging was performed. PET/CT showed mild to absent FDG uptake in the pulmonary nodules. Given the suspicion of metastasis, a fibroblast activation protein inhibitor (FAPI) PET/CT was subsequently performed, demonstrating intense Ga-68 FAPI uptake in a nodular lesion measuring 22 × 18 mm in the right lower lobe (SUVmax: 13.9).

Percutaneous core needle biopsy of the larger pulmonary nodule was performed. Histopathological examination confirmed metastatic SFT, characterized by spindle cells positive for CD34 and STAT6, with a Ki-67 proliferation index of 40% (Figure 1). Following evaluation by the Multidisciplinary Thoracic Oncology Board, wedge resection of both nodules was performed. Pathologic examination confirmed SFT with clear surgical margins.

The patient subsequently received four cycles of adjuvant chemotherapy consisting of ifosfamide and doxorubicin. The patient remained disease-free at 18 months of follow-up.

## Discussion

SFTs are rare mesenchymal neoplasms, and those originating from the prostate are even rarer [1,8]. Mesenchymal tumors account for less than 1% of all prostate neoplasms [9].

Similar to BPH, prostatic SFTs often present with lower urinary tract symptoms, including difficulty initiating urination and decreased urinary flow [10]. Accordingly, SFTs can easily be misdiagnosed as BPH. Our patient initially presented with the classic lower urinary tract symptoms.

A definitive diagnosis of SFT requires histopathological confirmation. A longer-than-expected duration of morcellation may increase the likelihood of findings other than BPH on pathology, as in our case. Microscopically, SFTs typically demonstrate alternating hypocellular and hypercellular areas, dense collagen deposition, and branching vascular patterns [11]. Immunohistochemical staining is critical for diagnosis; CD34, CD99, Bcl-2, and, more recently, nuclear STAT6 positivity are hallmarks of SFTs [6]. Strong diffuse staining for CD34 and STAT6 was observed in our patient, confirming the diagnosis.

Although certain histopathological features, such as tumor size >10 cm, high mitotic rate, necrosis, and nuclear pleomorphism, suggest malignancy, the biological behavior of SFTs remains unpredictable [5]. Notably, even tumors with benign histological features can eventually metastasize. In our case, despite a small tumor size (2 × 1.8 × 1.3 cm), low mitotic activity (0-1 mitoses/10 HPFs), and a low Ki-67 index (2%-3%), pulmonary metastases developed 23 months after diagnosis.

Owing to their potential for late recurrence or metastasis, extended follow-up is mandatory for patients with SFTs. Recurrences and distant metastases have been reported even two decades after initial treatment [7]. However, to our knowledge, no cases of metastatic SFT originating from the prostate have been previously described. Therefore, our case represents the first documented instance of metastatic prostatic SFT.

The management of metastatic SFTs has not yet been standardized. Surgical resection is the preferred treatment for isolated metastases. In our patient, both pulmonary nodules were completely excised via wedge resection. Given the high proliferative index of the metastatic lesions (Ki-67: 40%), adjuvant chemotherapy with ifosfamide and doxorubicin, which are traditionally used for soft tissue sarcomas, was administered.

## Conclusions

In conclusion, although extremely rare, SFT should be considered in the differential diagnosis of lower urinary tract symptoms, particularly when intraoperative findings are atypical. Radical excision, vigilance, and long-term follow-up are critical because of the unpredictable behavior of the tumor. Our case emphasizes the malignant potential of histologically benign prostatic SFTs and highlights the importance of awareness among urologists, oncologists, and pathologists regarding appropriate diagnosis and management.
